# A Protocol for the Development of the Intraoperative Complications Assessment and Reporting With Universal Standards Criteria: The ICARUS Project

**DOI:** 10.29337/ijsp.155

**Published:** 2021-08-06

**Authors:** Giovanni Cacciamani, Tamir Sholklapper, Rene Sotelo, Mihir Desai, Inderbir Gill

**Affiliations:** 1 USC Institute of Urology and Catherine and Joseph Aresty Department of Urology, Keck School of Medicine, University of Southern California, Los Angeles, CA, US

**Keywords:** intraoperative complications, intraoperative adverse event, protocol development, ICARUS, iAE checklist

## Abstract

**Introduction:**

Perioperative complications, especially intraoperative adverse events (iAEs), carry significant potential for long-term sequelae in a patient’s postoperative course. These events represent a substantial gap in contemporary surgical literature, with only a fraction of publications reporting intraoperative complications as outcomes of interest. To date, there is no universal standard for comprehensively reporting intraoperative complications in surgical practice and literature beyond the systems developed for grading individual events. We aim to establish a set of best-practice criteria for iAE reporting known as the **I**ntraoperative **C**omplication **A**ssessment and **R**eporting with **U**niversal **S**tandards (**ICARUS**) Guidelines.

**Methods and analysis:**

We will generate the ICARUS reporting guidelines using the EQUATOR Network development framework and the SQUIRE Guidelines. The initial step involves an umbrella review and meta-analysis of systemic reviews (SRs) assessing the perioperative adverse events of common surgeries. Measures for assessing, collecting, grading, and reporting the iAEs will be merged into a comprehensive list of criteria. Using a modified Delphi methodology, a team of expert surgeons (≥ 200 inpatient procedures/years) will contribute to and evaluate the proposed reporting guidelines. The panel will evaluate both the clinical usefulness and quality assessment and improvement utility of each criterion using a 5-point Likert. We expect multiple survey rounds until consensus regarding the utility of the guidelines is reached.

**Dissemination:**

We plan to share then validate the newly developed guidelines within each surgical field. Dissemination will involve publicly shared guidelines, simultaneous journal publications, conference presentations, encouragement for journal endorsement, and application for inclusion in the Equator Network database. The study team plans to continue collecting feedback for future extension of the intraoperative reporting guidelines.

**Highlights:**

- Intraoperative adverse events are underreported and lack homogeneity in surgical literature
- We aim to use a modified Delphi methodology to develop the consensus-based, intraoperative complications assessment and reporting with universal standards (ICARUS) guidelines
- We will disseminate the ICARUS guidelines through journal publications and presentations at national and international meetings; journals and professional organizations will be encouraged to endorse the ICARUS guidelines

## Introduction

Intraoperative adverse events (iAEs), carry significant potential for long-term consequences in a patient’s postoperative course. Without consistent and homogenous reporting, these events represent a substantial gap in contemporary surgical literature and clinical practice. By definition, an iAE is any unplanned incident related to a surgical intervention occurring between skin incision and skin closure [1]. Despite the availability of multiple intraoperative classification systems [1,2,3,4], the reporting of intraoperative adverse events remains exceedingly rare. Only a fraction of surgical publications reports intraoperative complications as outcomes of interest. An even smaller proportion of these publications appropriately report these events, demonstrating a marked heterogeneity in the literature [1,5,6,7].

Many reasons could be related to this dearth in iAE reporting, ranging from a lack of clear iAE definitions to a fear of litigation [8]. Broadly speaking, iAEs are negative outcomes, which, on the whole, epitomize a paradoxically well-documented bias in the literature [9]. More importantly, this gap in documentation limits the ability of surgeons and the medical community to assess and improve surgical quality directly. To date, there is no universal guideline for comprehensively reporting intraoperative complications in surgical practice and literature beyond the systems developed for grading individual events.

Herein, we report the protocol for developing the **I**ntraoperative **C**omplication **A**ssessment and **R**eporting with **U**niversal **S**tandards (**ICARUS**) Guidelines. We aim to establish a set of best-practice guidelines in iAE reporting for all surgical procedures in the clinical and academic setting. We recognize that dissemination and feasibility of these guidelines will be of equal importance in improving surgical research, and plan to address and evaluate both as a part of this initiative.

## Methods

**Figure 1:**
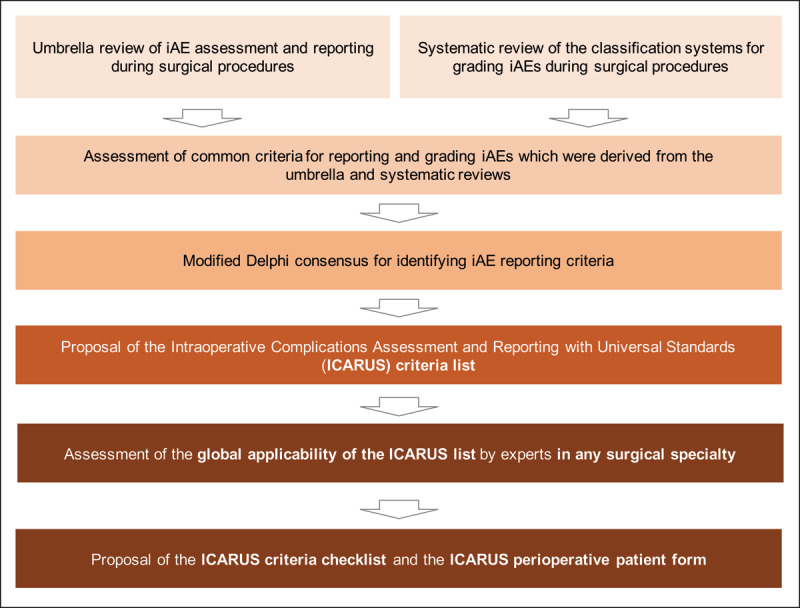
ICARUS study flowchart. iAEs: intraoperative adverse events.

Development of the ICARUS intraoperative adverse event reporting guidelines will involve three phases: criteria collection, modified Delphi consensus, and global validation (***Figure 1***). We plan to develop the reporting guidelines using the framework outlined by the EQUATOR Network (Enhancing the QUAlity and Transparency Of health Research; *www.equator-network.org/*) [10] and accordingly to the Standards for QUality Improvement Reporting Excellence (SQUIRE 2.0) Guidelines [11].

### Criteria collection

The initial step of the ICARUS project involves an umbrella review of systemic reviews (SRs) assessing the intraoperative adverse events of common oncological surgeries. Separately, a SR of iAE reporting and grading classifications will be carried out as previously reported [1]. For the umbrella review, the previously collected measures for assessing, collecting, grading, and reporting the iAEs will be merged and distilled into a comprehensive list of criteria by the study team. The aim of this first step is to collect the common criteria in the iAE reporting, used in the previous publications.

### Modified Delphi consensus

Using a modified Delphi methodology [12], a team of experienced surgeons (≥ 200 inpatient surgical procedure per year) affiliated with the AGILE consortium (Italian Group for Advanced Laparo-Endoscopic Surgery; *www.agilegroup.it*) will evaluate the reporting guidelines. The study team will not participate in the survey to avoid bias. The Delphi questionnaire will be administered via Google Forms (*https://docs.google.com/forms/*). In the first survey, panel members will outline their iAE reporting standards and objectively identify critical aspects of assessing, grading, and reporting iAEs. The study team will compile the responses and add them to the list created from the umbrella review. In subsequent surveys, the expert panel will evaluate both the clinical usefulness and quality assessment and improvement utility using a 1 to 5-point Likert scale with space provided for suggested edits and comments. Multiple rounds will be conducted until consensus is reached. After each round of Likert responses, the study team will calculate the agreement and distribution of responses. Likert responses will be dichotomized with positive values indicating agreement and neutral or negative values indicating disagreement. Internal consistency of the criteria will be assessed by Chronbach’s α [13]. Any criterion that achieves 30% or less agreement (i.e., fewer that 30%of respondents assess the criterion utility as 4 or 5) will be removed. Consensus will be reached once 70% agreement of panel members is achieved for clinical and quality improvement utility [1,14,15].

### Global validation

In the final phase of the ICARUS project, we plan to share then globally validate the newly developed guidelines into each surgical field (cardiothoracic surgery, colon and rectal surgery, general surgery, gynecology and obstetrics, gynecologic oncology, neurological surgery, ophthalmic surgery, oral and maxillofacial surgery, orthopaedic surgery, otorhinolaryngology, pediatric surgery, plastic and maxillofacial surgery, urology, and vascular surgery). Dissemination will involve publicly shared guidelines, journal publications, conference presentations, and application for inclusion in the Equator Network database. The study team plans to continue collecting feedback for future extension of the intraoperative reporting guidelines.
